# Supplementary figures and images for: Conformational heterogeneity of Savinase from NMR, HDX-MS and X-ray diffraction analysis
Source: PeerJ. 2020 Jun 26;8:e9408. doi: 10.7717/peerj.9408 (PMC7323712; doi:10.7717/peerj.9408)

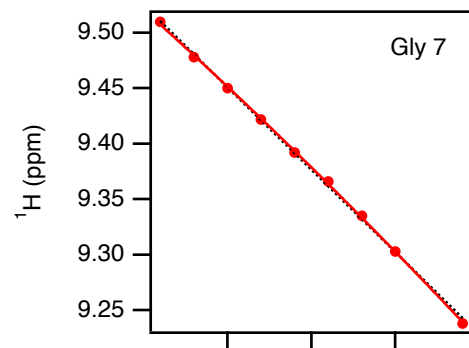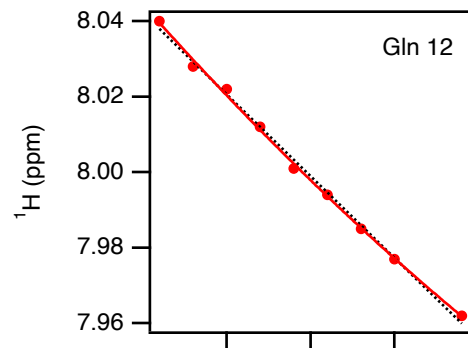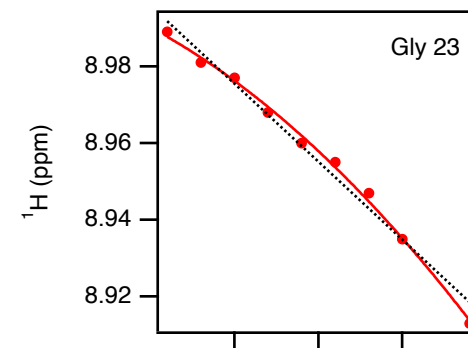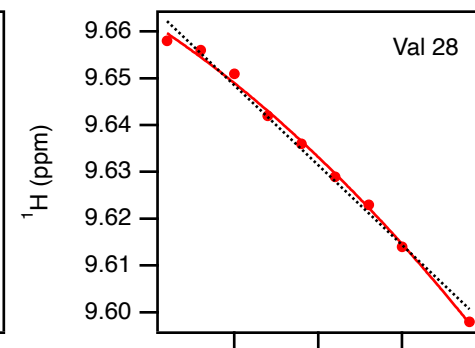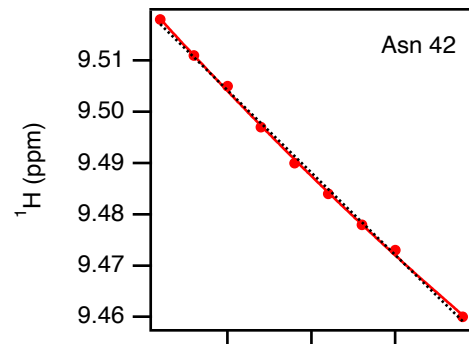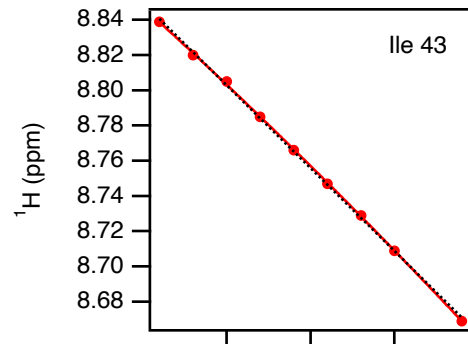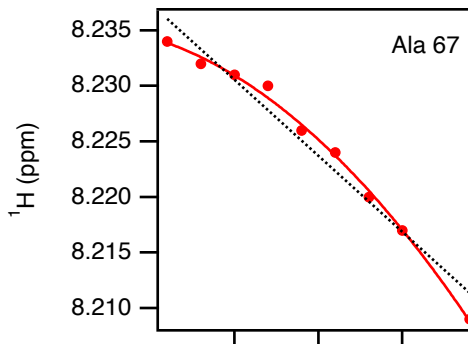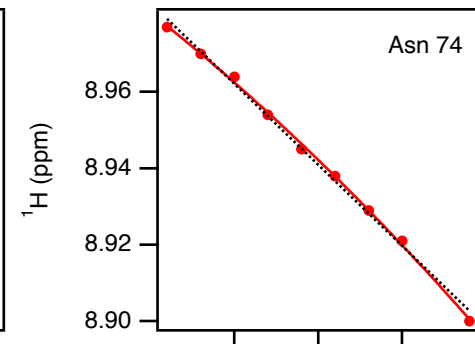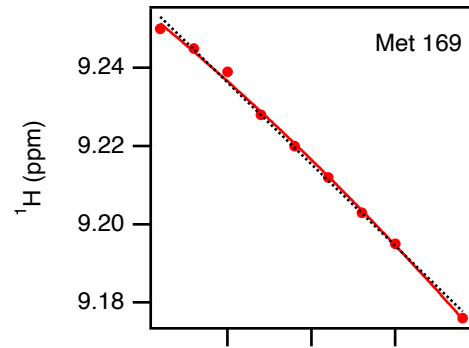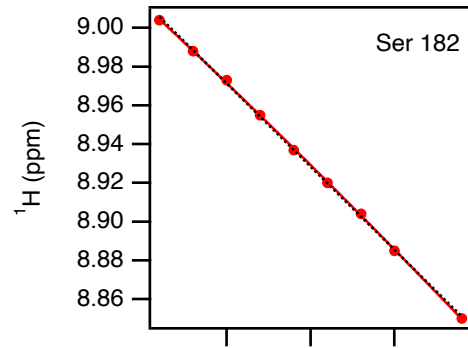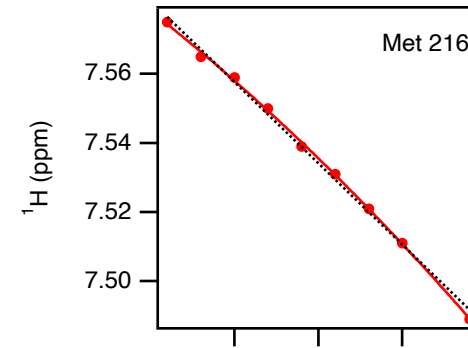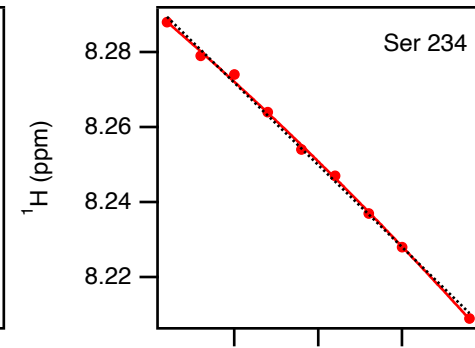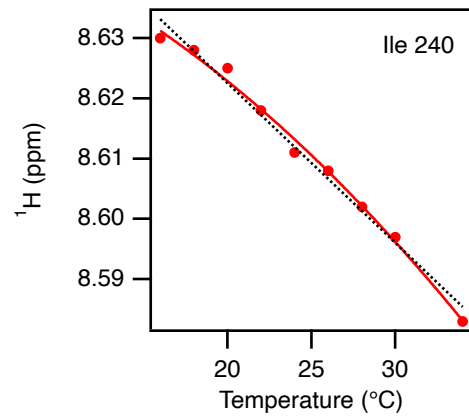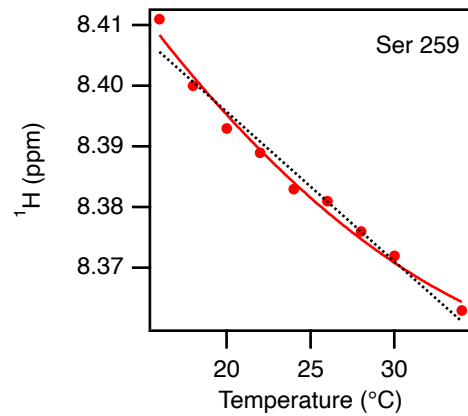

Supplement: Figure S1 — Only data for those residues for which the chemical shift changes are significantly better (P < 0.05, F test) represented by a second-order polynomial (solid line) than a first-order polynomial (dotted line) are shown. [file peerj-08-9408-s001.pdf]

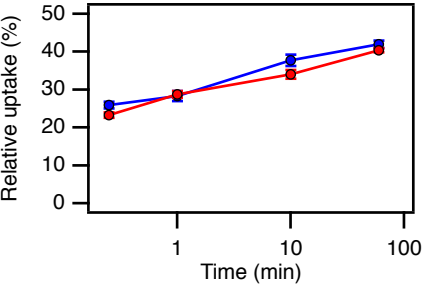

Supplement: Figure S2 — The deuterium content relative fully deuterated Savinase is plotted for uninhibited (red) and inhibited (blue) Savinase. Error bars indicate standard deviations for time points measured in duplicates. [file peerj-08-9408-s002.pdf]
