# Supplementary material for: Conformational heterogeneity of Savinase from NMR, HDX-MS and X-ray diffraction analysis
Source: PeerJ. 2020 Jun 26;8:e9408. doi: 10.7717/peerj.9408 (PMC7323712; doi:10.7717/peerj.9408)
Supplement: Figure S3 — Time dependent changes in deuterium content for all peptides analysed. Data for uninhibited and inhibited Savinase are show in grey and red, respectively. Maximum labelled controls are shown in black. Error bars indicate standard deviations for time points measured in replicates (n = 3). [file peerj-08-9408-s003.pdf]

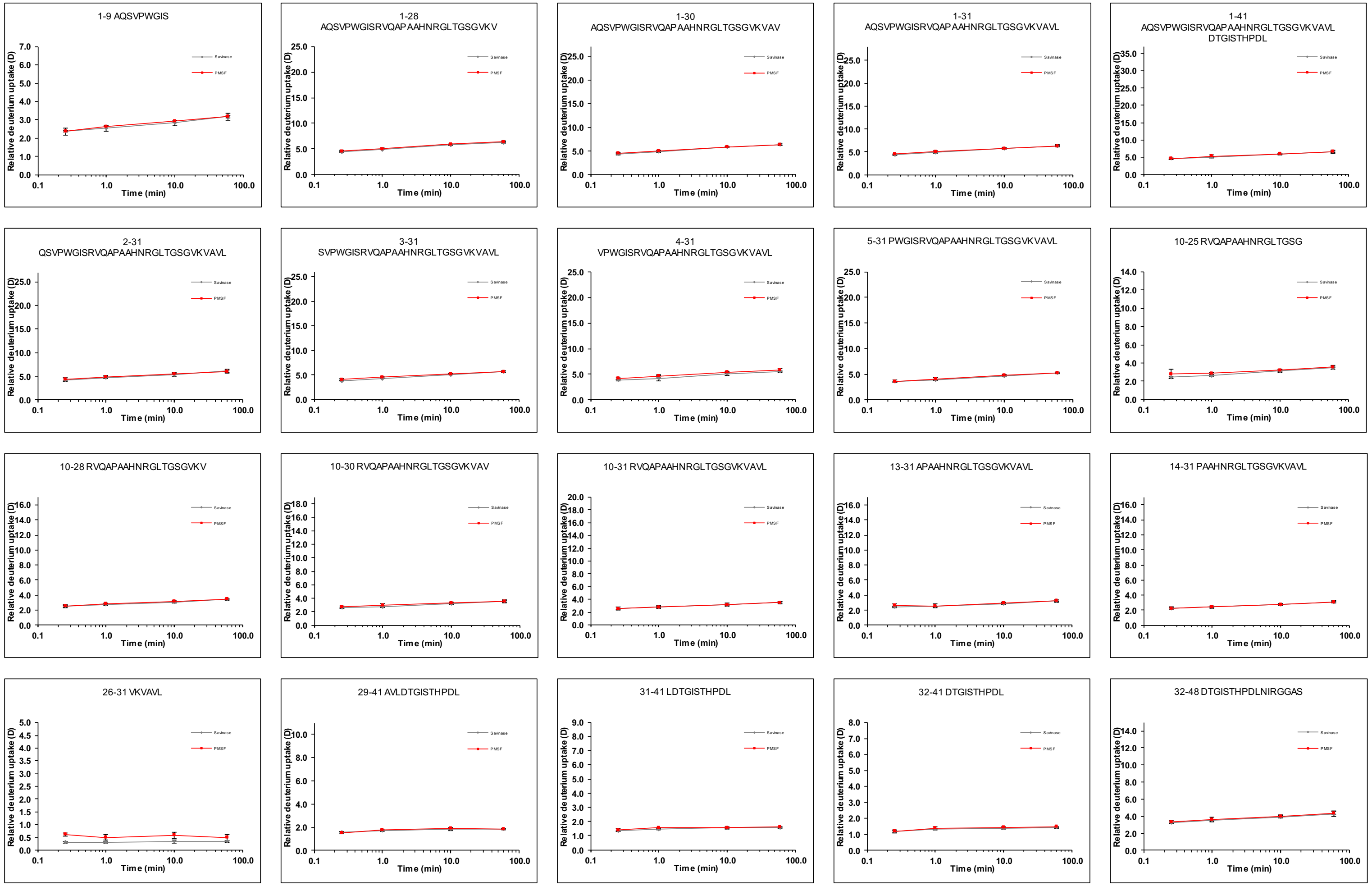

Figure S3 HDX-MS of Savinase. Time dependent changes in deuterium content for all peptides analysed. Data for uninhibited and inhibited Savinase are show in grey and red, respectively. Maximum labelled controls are shown in black. Error bars indicate standard deviations for time points measured in replicates (*n* = 3).

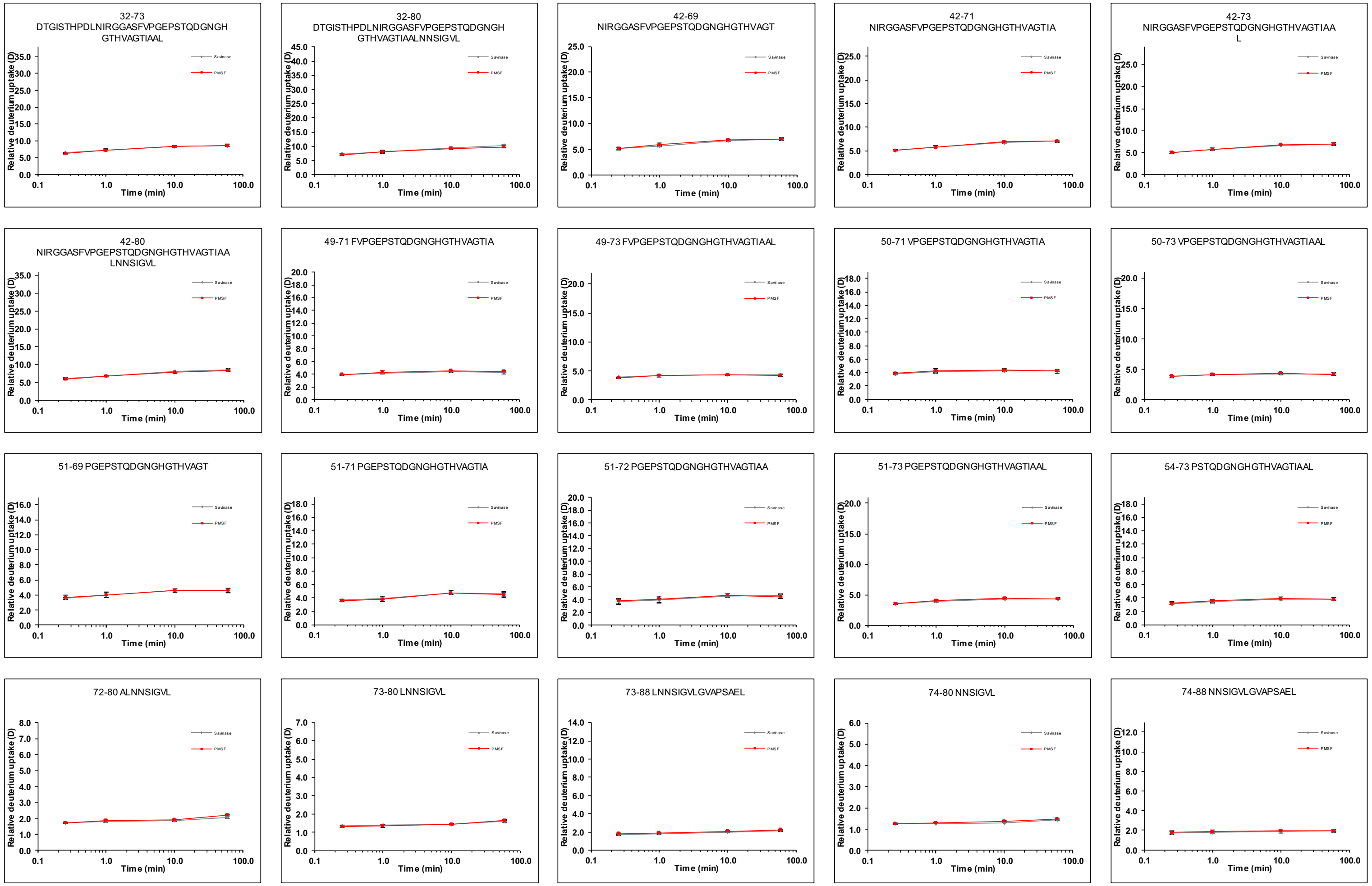
32-73 DTGISTHPDLNIRGGASFVPGEPSTQDGNGHGTHVAGTIAAL
32-80 DTGISTHPDLNIRGGASFVPGEPSTQDGNGHGTHVAGTIAALNNSIGVL
42-69 NIRGGASFVPGEPSTQDGNGHGTHVAGT
42-71 NIRGGASFVPGEPSTQDGNGHGTHVAGTIA
42-73 NIRGGASFVPGEPSTQDGNGHGTHVAGTIAAL
42-80 NIRGGASFVPGEPSTQDGNGHGTHVAGTIAALNNSIGVL
49-71 FVPGEPSTQDGNGHGTHVAGTIA
49-73 FVPGEPSTQDGNGHGTHVAGTIAAL
50-71 VPGEPSTQDGNGHGTHVAGTIA
50-73 VPGEPSTQDGNGHGTHVAGTIAAL
51-69 PGEPSTQDGNGHGTHVAGT
51-71 PGEPSTQDGNGHGTHVAGTIA
51-72 PGEPSTQDGNGHGTHVAGTIAA
51-73 PGEPSTQDGNGHGTHVAGTIAAL
54-73 PSTQDGNGHGTHVAGTIAAL
72-80 ALNNSIGVL
73-80 LNNSIGVL
73-88 LNNSIGVLGVAPSAEL
74-80 NNSIGVL
74-88 NNSIGVLGVAPSAEL
Relative deuterium uptake (D)
Time (min)
Savinase
PMSF

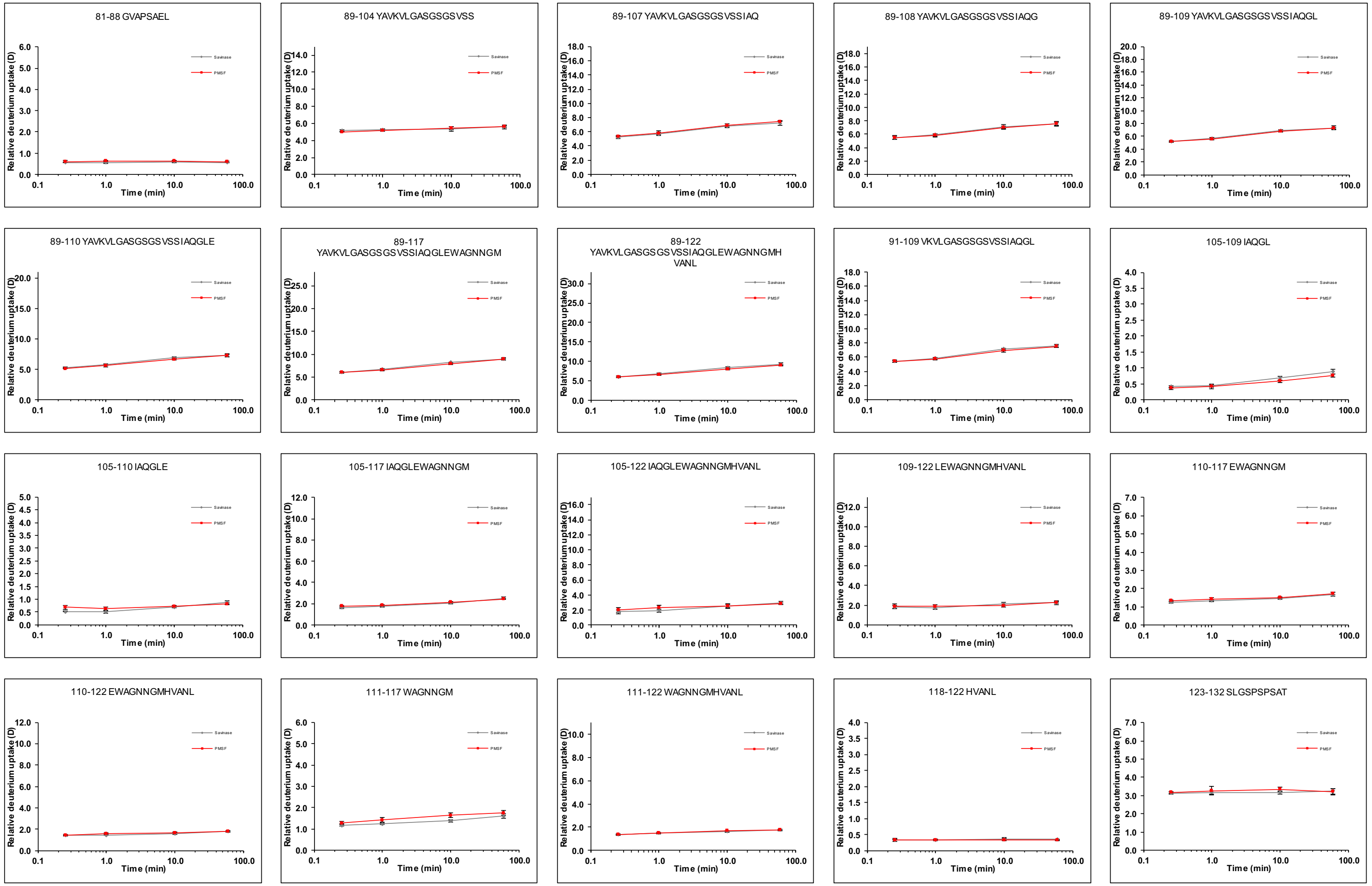
81-88 GVAPSAEL
89-104 YAVKVLGASGSGSVSS
89-107 YAVKVLGASGSGSVSSIAQ
89-108 YAVKVLGASGSGSVSSIAQG
89-109 YAVKVLGASGSGSVSSIAQGL
89-110 YAVKVLGASGSGSVSSIAQGLE
89-117 YAVKVLGASGSGSVSSIAQGLEWAGNNGM
89-122 YAVKVLGASGSGSVSSIAQGLEWAGNNGMHVANL
91-109 VKVLGASGSGSVSSIAQGL
105-109 IAQGL
105-110 IAQGLE
105-117 IAQGLEWAGNNGM
105-122 IAQGLEWAGNNGMHVANL
109-122 LEWAGNNGMHVANL
110-117 EWAGNNGM
110-122 EWAGNNGMHVANL
111-117 WAGNNGM
111-122 WAGNNGMHVANL
118-122 HVANL
123-132 SLGSPSPSAT
Relative deuterium uptake (D)
Time (min)
PMSF

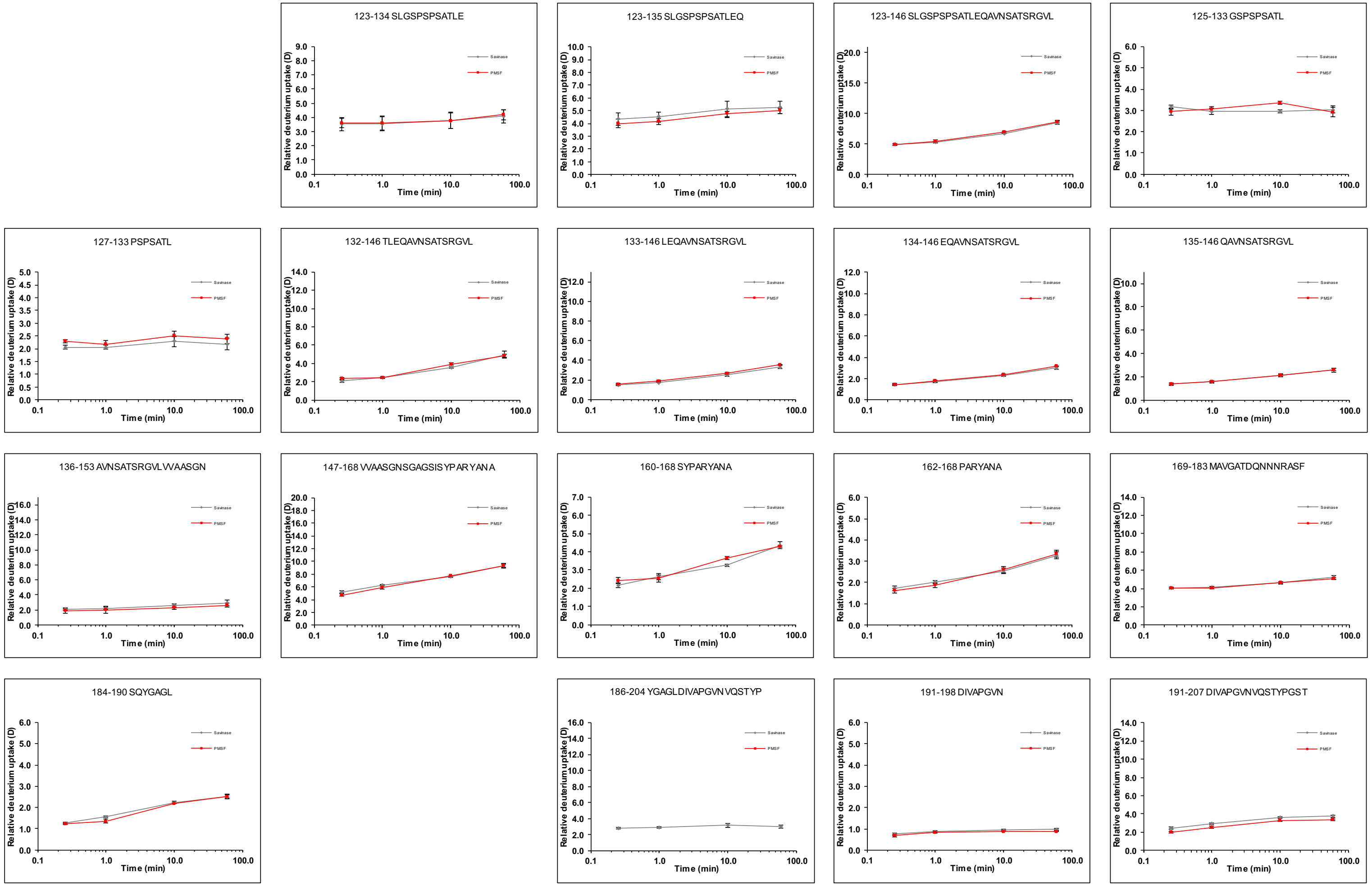
123-134 SLGSPSPSATLE
123-135 SLGSPSPSATLEQ
123-146 SLGSPSPSATLEQAVNSATSRGVL
125-133 GSPSPSATL
127-133 PSPSATL
132-146 TLEQAVNSATSRGVL
133-146 LEQAVNSATSRGVL
134-146 EQAVNSATSRGVL
135-146 QAVNSATSRGVL
136-153 AVNSATSRGVLVVAASGN
147-168 VVAASGNSGAGSISYPARYANA
160-168 SYPARYANA
162-168 PARYANA
169-183 MAVGATDQNNNRASF
184-190 SQYGAGL
186-204 YGAGLDIVAPGVNVQSTYP
191-198 DIVAPGVN
191-207 DIVAPGVNVQSTYPGST
Savinase
PMSF
Relative deuterium uptake (D)
Time (min)

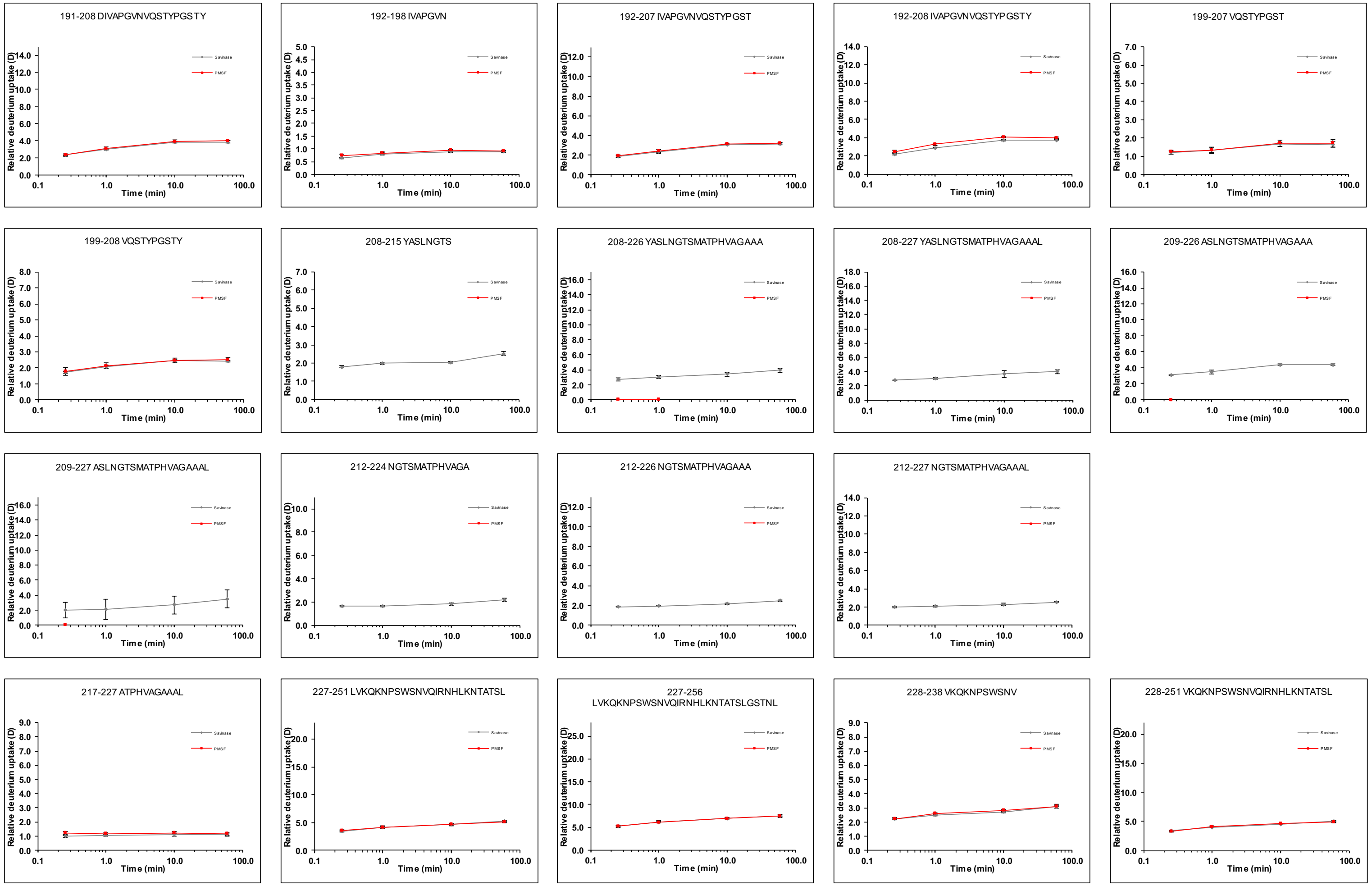
191-208 DIVAPGVNVQSTYPGSTY
192-198 IVAPGVN
192-207 IVAPGVNVQSTYPGST
192-208 IVAPGVNVQSTYPGSTY
199-207 VQSTYPGST
199-208 VQSTYPGSTY
208-215 YASLNGTS
208-226 YASLNGTSMATPHVAGAAA
208-227 YASLNGTSMATPHVAGAAAL
209-226 ASLNGTSMATPHVAGAAA
209-227 ASLNGTSMATPHVAGAAAL
212-224 NGTSMATPHVAGA
212-226 NGTSMATPHVAGAAA
212-227 NGTSMATPHVAGAAAL
217-227 ATPHVAGAAAL
227-251 LVKQKNPSWSNVQIRNHLKNTATSL
227-256 LVKQKNPSWSNVQIRNHLKNTATSLGSTNL
228-238 VKQKNPSWSNV
228-251 VKQKNPSWSNVQIRNHLKNTATSL
Relative deuterium uptake (D)
Time (min)
Savinase
PMSF

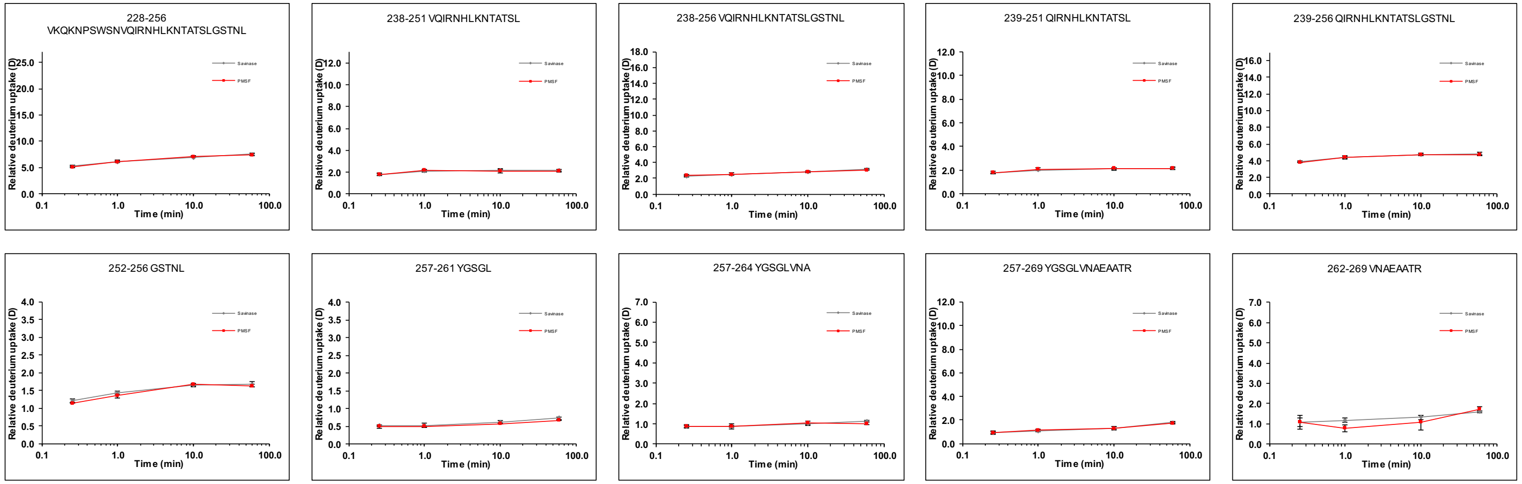
228-256 VKQKNPSWSNVQIRNHLKNTATSLGSTNL
238-251 VQIRNHLKNTATSL
238-256 VQIRNHLKNTATSLGSTNL
239-251 QIRNHLKNTATSL
239-256 QIRNHLKNTATSLGSTNL
252-256 GSTNL
257-261 YGSGL
257-264 YGSGLVNA
257-269 YGSGLVNAEAATR
262-269 VNAEAATR
Savinase
PMSF
Relative deuterium uptake (D)
Time (min)
