# Supplementary material for: Conformational heterogeneity of Savinase from NMR, HDX-MS and X-ray diffraction analysis
Source: PeerJ. 2020 Jun 26;8:e9408. doi: 10.7717/peerj.9408 (PMC7323712; doi:10.7717/peerj.9408)
Supplement: Table S3 [file peerj-08-9408-s006.pdf]

Table S3. Crystallographic statistics

**Data set**	RT	cryo
Data collection		
PDB code	6Y5T	6Y5S
Beamline, Diamond Light Source	I03	I04
Wavelength (Å)	0.88560	0.91474
Space group	$P2_12_12_1$	$P2_12_12_1$
Unit cell (a, b, c) (Å)	47.70, 62.205, 75.766	47.08, 62.03, 74.95
Resolution range (Å)	48.08 – 1.10	47.08 – 0.95
No of reflections collected	449,880 (21,456)	908,583 (39,594)
No. unique reflections	91,686 (4,525)	135791 (7,688)
Monomers in asymmetric unit	1	1
Completeness (%)	99.7 (99.9)	98.2 (94.8)
$\langle I/\sigma(I)\rangle$	10.8 (1.2)	13.7 (1.4)
$CC_{1/2}$	0.998 (0.502)	0.998 (0.605)
Multiplicity	4.9 (4.7)	6.7 (5.2)
R_{merge}	0.070 (1.341)	0.073 (1.075)
Refinement statistics		
Reflections for R_{free} (%)	4.92	4.98
R_{work} / R_{free} (%)	13.1 / 15.0	14.1 / 15.5
RMS deviations from idea geometry		
Bond length (Å)	0.006	0.005
Bond angles (°)	0.869	0.927
Chiral centres (Å^3)	0.081	0.088
Planar groups (Å)	0.006	0.006
Average B-factor (protein)	15.1	9.7
Ramachandran plot		
Preferred regions (%)	97.8	97.0
Allowed regions (%)	2.2	3.0
Outliers (%)	0	0

Statistics for the highest-resolution shells are shown in parentheses.
