# Supplementary material for: Conformational heterogeneity of Savinase from NMR, HDX-MS and X-ray diffraction analysis
Source: PeerJ. 2020 Jun 26;8:e9408. doi: 10.7717/peerj.9408 (PMC7323712; doi:10.7717/peerj.9408)
Supplement: Table S4 [file peerj-08-9408-s007.pdf]

Table S4. Amino acid residues in Savinase with alternative conformations in the RT and cryo crystals.

RT	Cryo
Ser3	Ser3
Ser9	
Thr22	
Ser24	
Ser36	Ser36
Thr37	
Pro39	
Asn42	
Ser55	
Ser76	Ser76
	Pro84
Tyr89	Tyr89
Ser103	Ser103
Ser104	Ser104
	Ser128
Pro129	Pro129
	Ser130
	Als131
	Thr132
Leu133	
Ser142	Ser142
Val148	
Ser158	Ser158
Ser160	Ser160
	Asn178
Val197	
Asn198	Asn198
Ser206	Ser206
Ser210	Ser210
Ans212	Asn212
Ser215	
	Lys231
Pro233	
	Val238
	Asn242
	Ser253
Tyr257	Tyr257
